# Pigment Intensity in Dogs is Associated with a Copy Number Variant Upstream of *KITLG*

**DOI:** 10.3390/genes11010075

**Published:** 2020-01-09

**Authors:** Kalie Weich, Verena Affolter, Daniel York, Robert Rebhun, Robert Grahn, Angelica Kallenberg, Danika Bannasch

**Affiliations:** 1 Department of Population Health and Reproduction, University of California-Davis, Davis, CA 95616, USA; kmweich@ucdavis.edu; 2 Department of Pathology, Microbiology, and Immunology, University of California-Davis, Davis, CA 95616, USA; vkaffolter@ucdavis.edu; 3 Department of Surgical and Radiological Sciences, University of California-Davis, Davis, CA 95616, USA; dyork@ucdavis.edu (D.Y.); rbrebhun@ucdavis.edu (R.R.); 4 Veterinary Genetics Laboratory, University of California-Davis, Davis, CA 95616, USA; ragrahn@ucdavis.edu (R.G.); akallenberg@ucdavis.edu (A.K.)

**Keywords:** canine, coat color, pheomelanin, eumelanin, dilution

## Abstract

Dogs exhibit a wide variety of coat color types, and many genes have been identified that control pigment production, appearance, and distribution. Some breeds, such as the Nova Scotia Duck Tolling Retriever (NSDTR), exhibit variation in pheomelanin pigment intensity that is not explained by known genetic variants. A genome-wide association study comparing light red to dark red in the NSDTR identified a significantly associated region on canine chromosome 15 (CFA 15:23 Mb–38 Mb). Coverage analysis of whole genome sequence data from eight dogs identified a 6 kb copy number variant (CNV) 152 kb upstream of *KITLG*. Genotyping with digital droplet PCR (ddPCR) confirmed a significant association between an increased copy number with the dark-red coat color in NSDTR (*p* = 6.1 × 10^−7^). The copy number of the CNV was also significantly associated with coat color variation in both eumelanin and pheomelanin-based Poodles (*p* = 1.5 × 10^−8^, 4.0 × 10^−9^) and across other breeds. Moreover, the copy number correlated with pigment intensity along the hair shaft in both pheomelanin and eumelanin coats. *KITLG* plays an important role in melanogenesis, and variants upstream of *KITLG* have been associated with coat color variation in mice as well as hair color in humans consistent with its role in the domestic dog.

## 1. Introduction

The dog, *Canis familaris,* was domesticated from wolves (*Canis lupus*), although the exact time and place are still under scientific debate [1,2,3,4]. Evidence for morphological variation between dogs and wolves is found through the study and dating of archeological remains; however, the colors of these animals are unknown. Although there is some color variation within wolves, with the majority of their coat colors being muted to enhance camouflage [5,6]. A black coat color variant was introduced into wolves from dogs, potentially providing an advantage for hunting at night [5]. Color variants that lighten or darken the coat have been identified as early as 10,000 years ago in archeological dog samples, indicating that color variation existed early in the history of dogs [6]. In the mid-16th century, paintings depicting dogs show the richness of coat colors that had been developed within domesticated dogs by that time. One example of this is “The Hunters in the Snow” by Pieter Bruegel the Elder (1565), which shows rich red-, brown-, and black-pigmented hunting dogs. Modern dog breeds were developed in the last couple of hundred years and many breeds exhibit striking pigment intensity compared to the more muted colors of their ancestors, the wolf.

The basis of the main coat color types in domestic dogs have been determined [7,8,9,10]. Similar to other mammals, there are yellow (pheomelanin) and black pigments (eumelanin) whose production is controlled by the pigment-type switching genes encoding melanocortin 1 receptor (*MC1R*) and agouti signaling protein (*ASIP*) [7]. MC1R is a G protein-coupled receptor expressed by melanocytes. Signaling by its ligand, the α-melanocyte-stimulating hormone (*MSH*), promotes eumelanin synthesis [11]. Disruption of MC1R signaling occurs when the ASIP protein is expressed, which leads to pheomelanin synthesis. In wolves, a pulse of ASIP expression occurs during hair growth, leading to banded hair shafts on the dorsum [8]. Variants that alter either ASIP or MC1R can affect the pheomelanin or eumelanin production. Solid yellow or red dogs have a loss of function mutation in *MC1R* that results in the sole production of pheomelanin pigment [8]. Both a recessive loss-of-function *ASIP* variant and a dominant gain-of-function variant in the gene encoding β-defensin 103 (*CBD103*) can result in black coat colors in dogs [7,9].

Genetic variants have been identified that modify the basic coat colors. Breeds with exclusive pheomelanin production with a white or light cream-colored coat are enriched for a missense variant in the *MFSD12* gene encoding major facilitator superfamily domain-containing 12 [12]. The dilution of pigment in some dogs can be the result of loss-of-function variants in the *MLPH* gene encoding melanophilin, resulting in abnormal clumping of pigment in the hair shaft and keratinocytes [13]. Brown coat color in dogs is an example of eumelanin lightening and is due to recessive alleles at *TYRP1*, which disrupt pigment production along the eumelanin pathway [14,15,16]. Pheomelanin appearance in dogs can vary from cream to deep red, with identical homozygous loss-of-function alleles at *MC1R* and *MFSD12* ([12] and results presented here), indicating that there are additional loci that can affect pigment intensity in dogs.

In this study, the genetic basis for light and dark red pheomelanin phenotypes was evaluated using a genome-wide association in the Nova Scotia Duck Tolling Retriever (NSDTR). By analyzing sequence coverage from whole genome sequence data, a previously identified copy number variant (CNV) on chromosome 15 upstream of the *KITLG* coding sequence was found to be associated with the red color intensity in the NSDTR [17]. Phenotype genotype correlation across a spectrum of coat colors and breeds demonstrates that this locus governs both eumelanin and pheomelanin pigment intensity across dog breeds. Quantitative analysis of color along the hair shaft shows that low pigment intensity leading to lighter color is due to less pigment at the base of the hair. Conversely, high pigment intensity occurs when the hair is uniformly pigmented. 

## 2. Materials and Methods

### 2.1. Sample Collection and DNA Extraction

Blood and saliva samples were collected from privately owned dogs through the University of California, Davis Veterinary Medical Teaching Hospital (VMTH) or submitted directly to the study from owners and veterinarians. Collection was performed under the supervision of the UC Davis Institutional Animal Care and Use Committee (protocol #20356). Genomic DNA from blood samples was extracted using the Qiagen Gentra Puregene Whole Blood Extraction Kit (QIAGEN, Valencia, CA, USA). Saliva samples were collected with Performagene Animal Collection swabs (DNA Genotek, Ottawa, ON, Canada) and extracted with the provided kit and protocol. Breed, date of birth, sex, and color were provided by owners.

### 2.2. Genome-Wide Association Study

Single-nucleotide variant (SNV) genotyping of 35 NSDTR was performed on the Illumina Canine HD 170 K BeadChip (Illumina, San Diego, CA, USA), which is mapped to the CanFam2.0 reference genome [18,19]. Dogs were categorized as light (*n* = 23) or dark (*n* = 13) based on visual inspection of their coat colors. The dogs were inspected in person or from photographs of the dogs in diffused lighting and graded by a single individual. Quality control and a Chi-square association analysis was performed with PLINK 1.9 [20,21]. All samples had a genotyping rate of at least 90%. In total, 63,746 SNVs were excluded due to a minor allele frequency of less than 5%, and 4238 SNVs were excluded due to a genotyping rate below 90%. Final analysis was performed with 105,678 SNVs, and the genome-wide significance was evaluated based on a Bonferroni threshold (*p ≤* 4.7 × 10^−7^). Figures were created with R packages qqman [22], GenAbel [23], and cgmisc [24].

### 2.3. Whole-Genome Sequencing

Whole-genome sequencing of 100 canine genomes was performed as described by Brown et al. [25]. Thirteen dogs were NSDTR (6 light, 6 medium, and 1 dark red) and 1 was an Irish Setter (dark red). The sequencing depth was an average 8.7× coverage per sample, and reads were aligned to the canine reference genome (CanFam 3.1) [26]. Variants from CanFam3.1 chr15: 23–38 Mb were screened for homozygous segregation with a light or dark coat color. Variants were screened for conservation across human, mice, and rats with the 4-Way Multiz Alignment and Conservation track on the UCSC genome browser CanFam2 (https://genome.ucsc.edu/index.html) [27,28,29,30]. Variant prediction for biological consequences was performed with the Ensembl Variant Effect Predictor (VEP) [31,32]. Coverage analysis across the region surrounding the CNV (chr15:29,800,000–29,850,000) was performed with igvtools in sliding 2 kb windows for each sample [33]. The average coverage excluding the 12 kb of the CNV was then used to normalize each sample to the reference genome and graphed in Microsoft Excel.

### 2.4. Digital Droplet PCR Genotyping

Copy number quantification of samples was performed with a digital droplet PCR (ddPCR) assay on a BioRad QX200 Droplet Digital System (BioRad, Hercules, CA, USA). Primers for the *KITLG* CNV were designed from the CanFam3.1 reference genome (F: GAGTAGGTGTAATTTACCGGACA, R: AGCTATTTGCACAGGCTTTTT, probe: CCCATCCATCTTTACCTTCAGAAACA) with a 5′ 6-FAM (fluorescein) dye and quenched with 3′ Zen Iowa Black dye as designed by Integrated DNA Technologies (IDT, Coralville, IA, USA). A control gene assay for ETS proto-Oncogene 1 (*ETS1*) was designed by BioRad with HEX (hexachloro-fluorescein) dye to a region conserved across mammalian Taxa chr5:6,166,572–6,166,747 (BioRad Assay ID: 10042961). Prior to PCR, DNA was digested with New England Biosciences AvaII restriction enzyme (Product #R0153L, NEB, Ipswich, MA, USA). The copy number was calculated using the BioRad QX200 software standard protocol as a ratio of positive KITLG droplets to positive *ETS1* droplets over all accepted droplets. Sample and primer concentrations were optimized to produce single templates per droplets. Statistical analysis of the results and generation of figures was performed with GraphPad Prism Version 8 (GraphPad, San Diego, CA, USA). A Mann-Whitney test was used to evaluate the differences between dark and light coat colors in NSDTRs and Poodles.

### 2.5. Quantitative Hair Pigment Analysis

In the Poodle, coat color information was provided by the owners (i.e., red, apricot, cream, white, black, silver, etc.). For breeds like the NSDTR, whose color variation is not recognized in the registered color of the dog, further quantification of the hair color from root to tip was performed by photographing the dogs’ coats under diffused light using a WhiBal G7 photography gray card (Michael Tapes Design, Melbourne, AUS). The photos were then color corrected and analyzed for the mean color depth at the root and tip of the hair shaft using the GNU Image Manipulation Program (GIMP version 2.10.8). The difference from root to tip was used to compare high and low copy number dogs and evaluated for statistical significance with a linear regression test in GraphPad Prism.

## 3. Results

### 3.1. Genome-Wide Association Study in Light and Dark Red NSDTR

**Figure 1:**
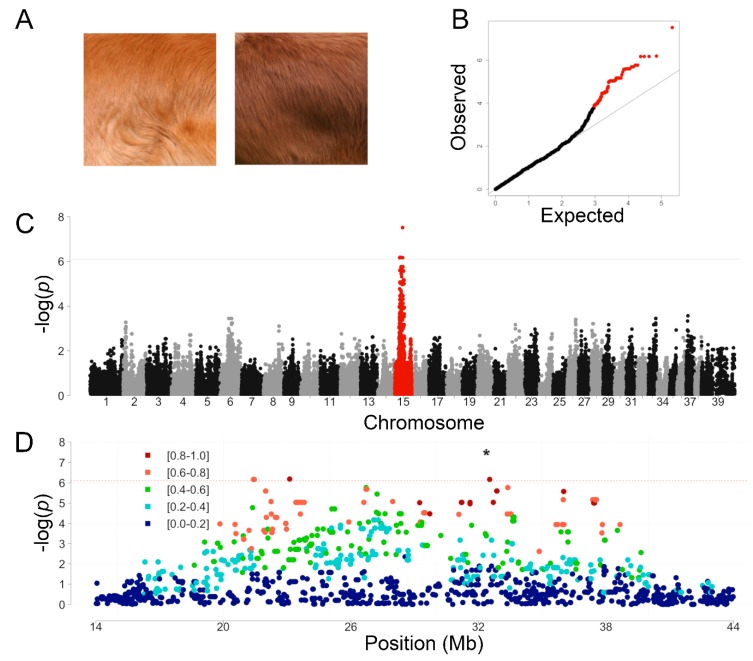
Genome-wide association coat color in the NSDTR. (**A**) Representative NSDTR pictures from light red (left) and dark red (right) coat-colored dogs. (**B**) Quantile-quantile plot from NSDTR GWAS showing a genomic inflation, λ, of 1.03. Chromosome 15 SNVs are highlighted in red. (**C**) Manhattan plot. A single locus on chromosome 15 (in red) reached genome-wide significance after a Bonferroni correction (CanFam2 chr15: 32,383,555, *p_raw_* = 3.09 × 10^−8^, *p_Bonferroni_* = 0.0033). Solid line indicates genome-wide significance based on Bonferroni multiple testing correction. (**D**) SNVs from a 30 Mb region surrounding the most associated SNV (indicated by *). SNVs are color coded by the *r*^2^ value to show linkage disequilibrium in the region. Red dashed line indicates the Bonferroni threshold.

The NSDTR breed comes in a spectrum of coat colors ranging from a light golden red to a dark coppery red (Figure 1A and AKC NSDTR breed standard). None of the previously identified canine coat color variants explain this phenotypic variation. A GWAS was performed to identify a region in the genome that is associated with this variation in coat color using 23 light red NSDTR and 13 dark red NSDTR. All dogs were wild type for the previously identified variant at *MFSD12* (orthologous to the human variant rs751,585,493 (ENST00000355415: Chr19:3,557,253 G > A (C > T in dogs); p.(Arg51Cys))) associated with lighter pheomelanin coat color [12]. One SNV was genome-wide significant with a *p_Bonferroni_* of <0.05 on CanFam2 chr15: 32,383,555 (CanFam3.1 chr15: 29,371,013) (*p_raw_* = 3.09 × 10^−8^, *p_Bonferroni_* = 0.0033) (Figure 1B). Analysis of the linkage disequilibrium (LD) identified a very large critical region on CanFam 2 chromosome 15 from 23 to 38 Mb (CanFam3.1 chr15: 20–35 Mb) with *r*^2^ values >0.8 with respect to the most associated SNV (Figure 1C).

### 3.2. Whole Genome Sequence Variant and Coverage Analysis

**Figure 2:**
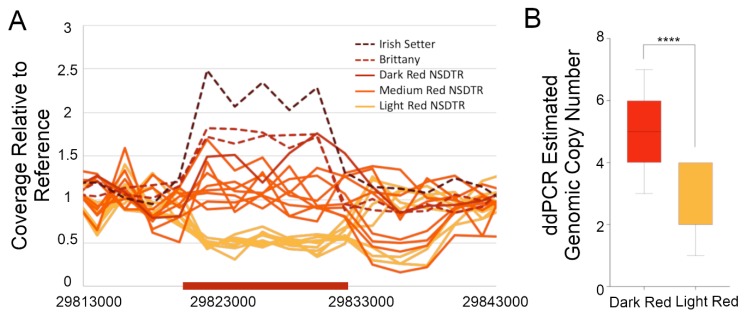
Relative coverage across the region of the *KITLG* CNV. (**A**) Relative coverage from paired-end whole-genome sequencing is plotted on the *y*-axis, and CanFam3.1 chr15 base pair position is plotted on the *x*-axis. The region of the CNV is marked by the red bar on the *x*-axis. Each line represents a single individual dog, and the color of the line represents the coat color of that dog. The solid lines represent NSDTR and the dashed lines represent Brittanys and an Irish Setter. (**B**) NSDTR comparison between light and dark dogs using digital droplet PCR to estimate the copy number. Comparison using a Mann–Whitney test. *p* = 6.1 × 10^−7^. Dark red, *n* = 32. Light red, *n* = 26. **** = highly significant.

In an initial screen for candidate causative variants, paired-end whole genome sequences of six light red NSDTR, one dark red NSDTR, and one dark red Irish Setter were investigated for variants in the critical region surrounding the best associated SNV. There were 81,560 SNV and 53,277 small insertion/deletion (indel) variants identified, and 112 of those variants segregated with coat color intensity (90 SNVs and 22 indels). None of these 112 variants were predicted to have protein coding effects, and none of the variants were in highly conserved regions of the genome. This region was also visually inspected on alignment files to screen for large indels or structural changes that were not identified by the variant calling software, and a literature search was performed to identify any known large structural variants in the region. Preliminary analysis of the aligned sequences of the abovementioned eight dogs plus an additional six medium red NSDTR showed that coverage over a previously identified 6 kb CNV at CanFam3.1 chr15:29,821,450–29,832,950 segregated with coat color, with dark red dogs showing an increase in the relative copy number compared to light red dogs (Figure 2A). Note that the reference genome shows two tandem copies of the CNV spanning a region of approximately 12 kb. The CNV is located in an intergenic region about 152 kb upstream from the closest gene, *KITLG*.

### 3.3. Validation of the KITLG CNV in Pheomelanin-Based Coat Colors

To validate the association of the high copy number at the *KITLG* CNV with the dark red coat color in the NSDTR, 26 dark red and 32 light red dogs confirmed to be homozygous for the loss of function alleles at *MC1R* and wild type for the cream-associated allele at *MFSD12* were genotyped at the CNV with a digital droplet PCR (ddPCR) assay. Dark red NSDTR (median = 5 genomic copies) had a significantly higher copy number of the CNV compared to light red NSDTR (median = 2 genomic copies, *p* = 6.1 × 10^−7^) (Figure 2B). After verifying an association in the NSDTR, the CNV was then genotyped in additional breeds with a fixed dark red phenotype. Irish Setters (*n* = 50), a breed characterized by intense dark red pigment, had a median genotype of eight genomic copies. Another breed with dark red pigment, the Brittany (*n* = 4), had a median genotype of seven genomic copies. 

**Figure 3:**
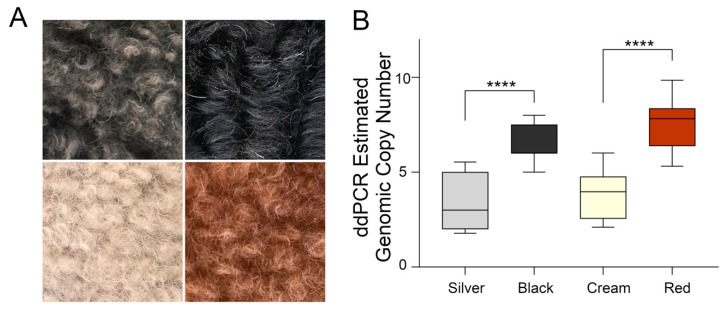
Eumelanin and pheomelanin-based Poodle coat colors and CNV genotypes. (**A**) Representative Poodle hair samples from eumelanin low intensity silver (top left), eumelanin high intensity black (top right), pheomelanin low intensity cream (bottom left), and pheomelanin high intensity red (bottom right). (**B**) Genomic copy number measured with digital droplet PCR from high and low intensity Poodles with pheomelanin and eumelanin-based coat colors. Silver compared to black (*p =* 1.5 × 10^−8^). Cream compared to red (*p* = 4.0 × 10^−9^). Sample numbers from left to right are as follows: *n* = 22, *n* = 25, *n* = 26, and *n* = 26. **** = highly significant.

In order to confirm the segregation of the high copy number with dark red coats, comparisons were made of cream- and red-colored Poodles (Figure 3A). the comparison of cream Poodles (*n* = 26) to red Poodles (*n* = 26) showed a significant increase in the copy number in red Poodles (median = 8 genomic copies) compared to cream (median = 3 genomic copies, *p* = 4.0 × 10^−9^) (Figure 3B). Cream dogs with a homozygous genotype for pheomelanin dilution at *MFSD12* (orthologous to human rs751,585,493: G > A) were not included in the analysis. Two other phenomenalistic breeds with non-functional MC1R that show coat color variation are the Golden Retriever (GR) and the yellow Labrador Retriever (LR). Their variation is not explained by the variant identified at *MFSD12* [12]. Neither of these breeds showed a significant association with the copy number and light or dark coat color (GR, *p* = 0.9977, light *n* = 8, medium *n* = 19, dark *n* = 8; LR, *p* = 0.7377, light *n* = 8, dark *n* = 8). The breeds and number of dogs of each coat color are shown in Table S1.

### 3.4. Validation of the KITLG CNV in Eumelanin-Based Coat Colors 

**Figure 4:**
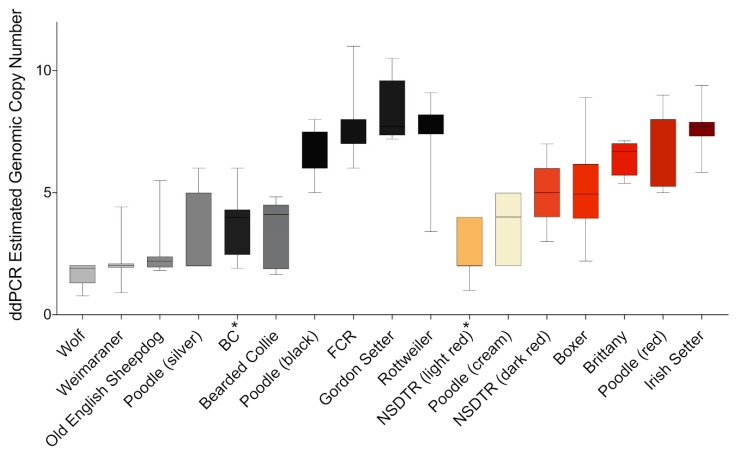
Survey of the digital droplet-estimated genomic copy number in common dog breeds and wolves. Breeds known to segregate light and dark coat colors in eumelanin and/or pheomelanin-based colors were separated by color. Breeds are color coded by an approximation of the overall coat color, and sample numbers are provided in Table S1. * Breeds whose copy number estimates did not seem to match their coat colors.

In the Poodle breed, pigment intensity differences occur in both pheomelanin and eumelanin-based coat colors (Figure 3A). Eumelanin-based colors range from silver or light grey to black, and these were evaluated for the CNV upstream of *KITLG*. Black Poodles (*n* = 25) had a significantly higher copy number (median = 6 genomic copies) compared to light grey (median = 2 genomic copies, *n* = 22) dogs (*p =* 1.5 × 10^−8^) (Figure 3B). Breeds fixed for light grey or black coat color were also genotyped, including light grey breeds like the Bearded Collie (median = 4 genomic copies, n = 5) and Old English Sheepdog (median = 2 genomic copies, *n* = 7), and black breeds like the Border Collie (BC, median = 4, *n* = 19), the Flat-Coated Retriever (FCR, median = 7, *n* = 25), and the Rottweiler (median = 7, *n* = 17). Additionally, grey wolves with unknown coat colors from North America (n = 2), Asia (*n* = 1), Europe (*n* = 1), and Africa (*n* = 1) were genotyped and all were found to have a very low copy number (approximately two genomic copies) (Figure 4). 

### 3.5. Comparison of the Hair Shaft in High and Low Copy Number Dogs

**Figure 5:**
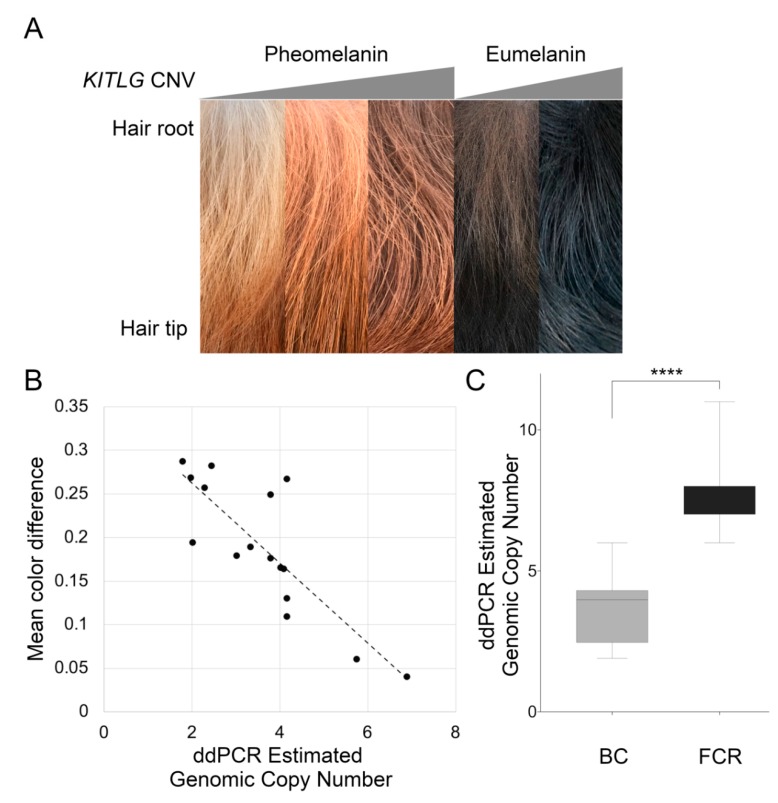
Hair sample quantification from pheomelanin and eumelanin-based breeds. (**A**) Representative hair samples from (left to right) low, medium, and high copy number NSDTR, and low and high copy number eumelanin-based dogs (BC and FCR). (**B**) Linear regression analyses revealed a significant association between an increased copy number and lower mean color difference between root and tip hair color in NSDTR (*p* = 0.00345, *n* = 17). (**C**) FCRs (*n* = 25) have a significantly higher copy number in comparison to BC (*n* = 19) in a ddPCR assay (*p* = 4.3 × 10^−8^). The graph color represents the approximate hair color at the root of the hair shaft. **** = highly significant.

Two breeds did not fit the association of the copy number with pigment intensity. The black and white BC did not have a high copy number as expected, and NSDTR with a low copy number were not as light colored as low copy number pheomelanin-based Poodles. Poodles have mutations that alter their hair length, which functions by extending the anagen phase of the hair growth cycle. This led us to hypothesize that the pigment intensity might be due to changes along the length of the hair in dog breeds with more typical anagen phase growth. Changes in the pigment color along the hair shaft were evaluated quantitatively using high-resolution photographs and compared between high and low copy number dogs. Low copy number dogs appear to have less pigmentation at the root compared to high copy number dogs (Figure 5A). Linear regression analysis of 17 NSDTR with the mean color difference and estimated ddPCR genomic copy number identified a significant association of the high copy number with a low mean color difference (*p* = 0.0035) (Figure 5B). Additionally, low copy number eumelanin-based breeds showed a higher mean color difference compared to high copy number eumelanin-based breeds, such as the FCR (*p* = 4.3 × 10^−8^) (Figure 5C).

## 4. Discussion

Segregation of pheomelanin intensity in the NSDTR enabled identification of a region on CFA 15 associated with pigment intensity in dogs. Variant analysis identified a 6 kb CNV upstream of *KITLG* that was highly associated with pheomelanin intensity within the NSDTR (*p* < 0.0001) and in other breeds. The copy number of this CNV is significantly associated with pheomelanin and eumelanin intensity in the Poodle and across breeds. All tested wolves had two copies of this element, indicating that chromosomes with higher copy numbers and high pigment intensity represent a derived state. Pigment intensity is due to the differing distribution of pigment along the hair shaft, with low copy number individuals having lighter hair at the root while more intensely pigmented individuals have no difference in pigment intensity from the root to hair tip. In the Poodle breed, the hair is continuously growing, such that the difference in colors associated with the different CNV alleles are particularly striking. 

*KITLG* and its receptor, *KIT*, have previously been shown to function in hematopoiesis, melanogenesis, and gametogenesis [34]. Pleiotropic effects of genes responsible for coat color variation are common in mammals [35]. In this case, this ligand receptor pair is essential for stem cell populations to migrate and proliferate during development [36,37]. These stem cell populations include the primordial germ cells, hematopoietic progenitor cells, and melanoblasts. In addition to this early developmental function in melanoblast migration, *KITLG* is important for postnatal cutaneous melanogenesis [38]. Specifically, *KITLG* expression is essential in hair follicle epithelial cells for melanocyte terminal differentiation [39]. The essential role that *KITLG* plays in melanogenesis, both developmentally and in the hair shaft, is consistent with its identification as a pigmentation intensity locus in dogs.

Variants of *KITLG* and its receptor KIT cause pigmentation alternations in humans. Coding sequence variants in *KITLG* have been shown to cause familial progressive hyperpigmentation with or without hypopigmentation [40] as well as Waardenburg syndrome type 2 (with pigmentary abnormalities) [41]. Additionally, coding variants within the receptor KIT have been associated with classic autosomal dominant piebaldism characterized by congenital patches of skin with no melanocytes present [42,43,44,45]. However, regulatory variants have been shown to affect pigment intensity. Sulem et al. (2007) found that a variant upstream of *KITLG* was associated with hair color in a genome-wide association scan in Icelanders and Dutch [46]. This variant likely lies in linkage disequilibrium, with an extended haplotype upstream of *KITLG* showing a strong signature of selection in humans [46,47,48]. Guenther et al. (2014) found that this region drove expression exclusively in hair follicles using reporter constructs in mice [49]. *KITLG* has diverse important developmental functions; however, the alternation of just hair follicle expression may be a means to limit the effects to pigmentation phenotypes.

Regulatory variants of *KITLG* (or *Kitl* in the mouse) have been identified as causing pigment differences in other animal species. The original *Kitl* mouse mutants were called steel due to their uniformly diluted coat colors [50]. The steel panda allele is caused by an inversion that disrupts the upstream regulatory region of *Kitl*, which also leads to diluted pigmentation [49,51]. In both goats and mink, *KITLG* expression is lower in lighter-colored animals as compared to more darkly pigmented animals [52,53]. In this work, expression levels of *KITLG* were not evaluated due to challenges in obtaining skin samples from healthy pet dogs; however, based on the work done in other species, the CNV likely alters expression levels of *KITLG* in the hair follicle.

While many dog breeds tested had a pigment intensity correlated to the *KITLG* CNV, some breeds with pheomelanin variation did not. Notably, the Golden Retriever and Labrador Retriever had variability at the CNV, but it did not correlate to their coat colors, indicating that there are still additional pigment intensity loci or variants to be identified in dogs. It is not surprising that many loci affect pigmentation since dogs are under such strong artificial selection for the variety in coat colors.

The canine CNV upstream of *KITLG* was previously associated with susceptibility for digital squamous cell carcinoma (DSCC) in darkly colored dogs [54]. The analysis was performed in darkly pigmented cases of DSCC and control dogs, and a high copy number of this CNV was identified as the susceptibility locus; however, this effect was only found in eumelanistic cases with functional *MC1R*. *KITLG* variants have been implicated in melanoma, testicular cancer, colorectal cancer, and general cancer risk in humans [55,56,57,58]. The risk allele for DSCC in dogs had >four copies of the CNV and homozygous animals were more likely to have DSCC [54]. Black-pigmented dogs are highly overrepresented for DSCC, as 92% of samples were from black-coated dogs [59]. However, there are breeds like the Flat-Coated Retrievers that have a high copy number, black hair coat, and are not predisposed to this type of tumor [59]. It is possible that a high copy number at the *KITLG* CNV is another risk factor for breeds that are already more susceptible to this particular tumor type. Alternatively, some black dogs with high *KITLG* copy numbers may also have protective alleles. 

Regulatory variants of human *KITLG* that affect hair color are located in a similar region to the CNV identified in dogs. Conservation tracks within the CNV are 86.1% identical to the human sequence. The human homologous region to the canine CNV is located 200 kb upstream of human *KITLG*. The strongest selective signature for *KITLG* in humans lies 218 kb upstream of the coding sequence [60] while the SNVs associated with light pigmentation are located 355 kb upstream of *KITLG* [47]. In dogs, there is also evidence that *KITLG* is under selective pressure as it was one of the top 20 regions of the genome to have a signature of selection within domestic dogs [61,62]. Interestingly, in canines, the selection by humans is for darker pigmentation over the wild-type wolf colors. Vivid and rich pigmentation within domestic dogs is one way that they may have been distinguished from wolves. Wolves have just a single copy of this 6 kb region, indicating that amplification of the region occurred within domestic dogs. It is interesting to speculate that this may have been one useful means of distinguishing partner proto-dogs from their wild ancestors.

## 5. Conclusions

The *KITLG* CNV is associated with coat pigment intensity in the domestic dog. An increase in the copy number is associated with a darker, more intense appearance to the coat and a more uniform pigment intensity across the hair shaft in many breeds. Wolves do not show copy number variation at this locus, and previous genome-wide screens have identified the *KITLG* CNV region as being under selection in domestic dogs. *KITLG* plays an important role in melanogenesis, both in development and in the adult hair follicle, and genetic variants upstream of *KITLG* in both mice and humans are associated with coat and hair color variation. This research suggests that the *KITLG* CNV is a new intensity locus in domestic dogs.

## Supplementary Materials

The following are available online at https://www.mdpi.com/2073-4425/11/1/75/s1, Table S1: Number of dogs genotyped for KITLG CNV copy number on ddPCR.
