# Correction: Weich, K., et al. Pigment Intensity in Dogs Is Associated with a Copy Number Variant Upstream of *KITLG*. *Genes* 2020, *11*, 75

**DOI:** 10.3390/genes12030357

**Published:** 2021-03-01

**Authors:** Danika L. Bannasch, Verena K. Affolter, Daniel York, Robert B. Rebhun, Robert A. Grahn, Kalie M. Weich, Angelica Kallenberg

**Affiliations:** 1 Department of Population Health and Reproduction, University of California-Davis, Davis, CA 95616, USA; kmweich@ucdavis.edu; 2 Department of Pathology, Microbiology, and Immunology, University of California-Davis, Davis, CA 95616, USA; vkaffolter@ucdavis.edu; 3 Department of Surgical and Radiological Sciences, University of California-Davis, Davis, CA 95616, USA; dyork@ucdavis.edu (D.Y.); rbrebhun@ucdavis.edu (R.B.R.); 4 Veterinary Genetics Laboratory, University of California-Davis, Davis, CA 95616, USA; ragrahn@ucdavis.edu (R.A.G.); akallenberg@ucdavis.edu (A.K.)

The authors wish to make the following corrections to this paper [1] due to errors identified after publication. The authorship order has been changed to reflect the appropriate contribution to this version of the manuscript. The Author Contributions have been changed as follows: K.B. has been removed from Data curation, Formal analysis, Writing-original draft and Writing- review and editing. D.B. has been added to Visualization.

The *p* values have been changed from NSDTR (*p* = 6.1 × 10^−7^) to (*p* = 4.1 × 10^−11^) and “both eumelanin and pheomelanin-based Poodles (*p* = 1.5 × 10^−8^, 4.0 × 10^−9^)” to “eumelanin-based Poodles (*p* = 2.1 × 10^−11^)”.

## 2. Materials and Methods

### 2.5. Quantitative Hair Pigment Analysis

A new methods description has been added. “The photos were then color corrected and analyzed for the mean color depth at the root and tip of the hair shaft using the GNU Image Manipulation Program (GIMP version 2.10.8). The difference from root to tip was used to compare high and low copy number dogs and” was replaced with this “The color intensity was measured using Adobe photoshop in triplicate over 50 pixels at the root and tip in a single photograph. The mean intensity of root/tip was compared to the ddPCR results and”.

## 3. Results

### 3.2. Whole Genome Sequence Variant and Coverage Analysis

Figure 2 has been replaced. Values in the figure legend have been changed from “*p* = 6.1 × 10^−7^. Dark red, *n* = 32. Light red, *n* = 26” to “*p* = 4.1 × 10^−11^. Dark red, *n* = 28. Light red, *n* = 34”.

### 3.3. Validation of the KITLG CNV in Pheomelanin-Based Coat Colors

Paragraph 1

The number of NSDTR was changed from 26 to 28 dark red and 32 to 34 light red. The *p* value was changed from *p* = 6.1 × 10^−7^ to *p* = 4.1 × 10^−11^. The number of Irish setters was changed from 50 to 48 and the median number of genomic copies from eight to seven. The median number of genomic copies in Brittany was changed from seven to six.

Paragraph 2

Cream was changed to white. These sentences “The comparison of cream Poodles (*n* = 26) to red Poodles (*n* = 26) showed a significant increase in the copy number in red Poodles (median = 8 genomic copies) compared to cream (median = 3 genomic copies, *p* = 4.0 × 10^−9^) (Figure 3B). Cream dogs with a homozygous genotype for pheomelanin dilution at *MFSD12* (orthologous to human rs751,585,493: G > A) were not included in the analysis.” were changed to this “The comparison of white Poodles (*n* = 21) to red Poodles (*n* = 29) showed an increase in the copy number in red Poodles (median = 8 genomic copies) compared to white (median = 7 genomic copies, *p* = 0.003); however it was not statistically significant when white dogs with a homozygous genotype for pheomelanin dilution at *MFSD12* (orthologous to human rs751,585,493: G > A) were excluded from the analysis (Figure 3B)”.

Figure 3 was replaced.

The last sentence of the legend was changed from “Silver compared to black (*p* = 1.5 × 10^−8^). Cream compared to red (*p* = 4.0 × 10^−9^). Sample numbers from left to right are as follows: *n* = 22, *n* = 25, *n* = 26, and *n* = 26.” to “Silver compared to black was significant (*p* = 1.5 × 10^−8^), while white compared to red was not. Sample numbers from left to right are as follows: *n* = 27, *n* = 425, *n* = 21, and *n* = 29”.

### 3.4. Validation of the KITLG CNV in Eumelanin-Based Coat Colors

The number of black Poodles was changed from *n* = 25 to *n* = 42. The number of grey Poodles was changed to *n* = 27 and the *p*= 2.1 × 10^−11^. The numbers of dogs and the mean copy number were changed as follows; “Border Collie (BC, median = 3, *n* = 26), the Flat-Coated Retriever (FCR, median = 7, *n* = 20), and the Rottweiler (median = 8, *n* = 17)” from “Border Collie (BC, median = 4, *n* = 19), the Flat-Coated Retriever (FCR, median = 7, *n* = 25), and the Rottweiler (median = 7, *n* = 17)”.

Figure 4 was replaced.

### 3.5. Comparison of the Hair Shaft in High and Low Copy Number Dogs

This sentence “The black and white BC did not have a high copy number as expected, and NSDTR with a low copy number were not as light colored as low copy number pheomelanin-based Poodles.” was replaced with this “The black and white BC did not have a high copy number as expected, and white Poodles had higher copy numbers than expected, likely due to the presence of other mutations that affect their pheomelanin intensity. Eumelanin-based Poodles did have a strong correlation of copy number and color”.

The last two sentences of the paragraph also had updated numbers and *p* values. This “Linear regression analysis of 17 NSDTR with the mean color difference and estimated ddPCR genomic copy number identified a significant association of the high copy number with a low mean color difference (*p* = 0.0035) (Figure 5B). Additionally, low copy number eumelanin-based breeds showed a higher mean color difference compared to high copy number eumelanin-based breeds, such as the FCR (*p* = 4.3 × 10^−8^) (Figure 5C).” was replaced by this: “Linear regression analysis of 15 NSDTR with color intensity ratios from root to tip and estimated ddPCR genomic copy number identified a significant association of the high copy number with a low ratio (*p* = 0.0022) (Figure 5B). Additionally, a eumelanin breed with light roots (Border Collie) had a significantly lower copy number compared to uniformly pigmented Flat-Coated Retrievers FCR (*p* = 2.3 × 10^−7^) (Figure 5C)”.

Figure 5B,C were replaced and the legends were modified accordingly.

## 4. Discussion

The third sentence was changed from “The copy number of this CNV is significantly associated with pheomelanin and eumelanin intensity in the Poodle and across breeds.” to “The copy number of this CNV is significantly associated with eumelanin intensity in the Poodle and across breeds and, to a lesser extent, pheomelanin”.

A sentence was added to the 5th paragraph “In addition, white Poodles likely have multiple genes, including the *KITLG* CNV and *MFSD12* [12] involved with contributing to their light phenotype”.

Table S1 was corrected consistent with these changes.

The authors apologize for any inconvenience caused and state that the scientific conclusions are unaffected. The published version will be updated on the article webpage, with a reference to this correction notice.

## References

[1] Weich K., Affolter V., York D., Rebhun R., Grahn R., Kallenberg A., Bannasch D. (2020). Pigment Intensity in Dogs is Associated with a Copy Number Variant Upstream of KITLG. Genes.

