# Supplementary material for: Pigment Intensity in Dogs is Associated with a Copy Number Variant Upstream of KITLG
Source: Genes (Basel). 2020 Jan 9;11(1):75. doi: 10.3390/genes11010075 (PMC7017362; doi:10.3390/genes11010075)
Supplement: Supplementary file 1 [file genes-11-00075-s001.pdf]

Table 1. Number of dogs genotyped for *KITLG* CNV copy number on ddPCR.

Breed	Color	N on ddPCR
Bearded Collie	Grey and white	5
Border Collie	Black and white	19
Boxer	Any	16
Brittany	Red and white	4
Flat Coated Retriever	Black	25
Golden Retriever	Light Golden	8
Golden Retriever	Medium Golden	19
Golden Retriever	Dark Golden	8
Gordon Setter	Black and tan	6
Irish Setter	Red	53
Labrador Retriever	Light Yellow	8
Labrador Retriever	Dark Yellow	8
Nova Scotia Duck Tolling Retriever	Dark Red	26
Nova Scotia Duck Tolling Retriever	Light Red	32
Nova Scotia Duck Tolling Retriever	Other	44
Old English Sheepdog	Grey and white	7
Poodle	Cream	26
Poodle	Red	26
Poodle	Black	22
Poodle	Silver	25
Poodle	Other	29
Rottweiler	Black and tan	20
Weimaraner	Grey	24
Wolf	Unknown	5
